# The Transmission of Intergenerational Epigenetic Information by Sperm microRNAs

**DOI:** 10.3390/epigenomes6020012

**Published:** 2022-04-07

**Authors:** Grace S. Lee, Colin C. Conine

**Affiliations:** 1 Pharmacology Graduate Group, University of Pennsylvania Perelman School of Medicine, Philadelphia, PA 19104, USA; gracesun@pennmedicine.upenn.edu; 2 Departments of Genetics and Pediatrics—Penn Epigenetics Institute, Institute of Regenerative Medicine, and Center for Reproduction and Women’s Health, University of Pennsylvania Perelman School of Medicine, Philadelphia, PA 19104, USA; 3 Division of Neonatology, Children’s Hospital of Philadelphia, Philadelphia, PA 19104, USA

**Keywords:** microRNAs sperm, epigenetic inheritance, environmental exposures

## Abstract

Epigenetic information is transmitted from one generation to the next, modulating the phenotype of offspring non-genetically in organisms ranging from plants to mammals. For intergenerational non-genetic inheritance to occur, epigenetic information must accumulate in germ cells. The three main carriers of epigenetic information—histone post-translational modifications, DNA modifications, and RNAs—all exhibit dynamic patterns of regulation during germ cell development. For example, histone modifications and DNA methylation are extensively reprogrammed and often eliminated during germ cell maturation and after fertilization during embryogenesis. Consequently, much attention has been given to RNAs, specifically small regulatory RNAs, as carriers of inherited epigenetic information. In this review, we discuss examples in which microRNAs have been implicated as key players in transmitting paternal epigenetic information intergenerationally.

## 1. Introduction

While there are multiple contrasting definitions of “epigenetics,” one definition is the heritability of a phenotype without any change to the inherited genetic makeup (DNA) of the cell or organism. Expanding on that definition, epigenetic inheritance occurs when a phenotype is transmitted from one generation to the next without an underlying genetic change to the inherited DNA. For epigenetic inheritance to occur, information must be programmed into the germline and gametes of parents and then propagated through fertilization and early development to ultimately transmit a phenotype to offspring. Although the mechanism of this transfer of information is poorly understood, instances of intergenerational inheritance through epigenetic means have been increasingly identified over the last 10–15 years through studying the effects of the parental environment on offspring phenotype.

It has become progressively more appreciated that environmental conditions experienced by parents, including various types of stress, diet, and chemical exposures, can result in non-genetically inherited phenotypes in offspring. Several epidemiological studies in humans have attempted to demonstrate non-genetically inherited phenotypes resulting from the ancestral environment. One case, known as the Överkalix cohort, found that the food available to men was correlated with increased susceptibility to obesity and cardiovascular disease in male progeny two generations later. Similarly, women and their granddaughters displayed the same trend. In both cases, when grandparents had limited food accessibility in their early adulthood, their grandchild presented with increased susceptibility to disease and mortality [1,2]. In another example, sons (but not daughters) of Civil War prisoners of war (POWs) exhibited increased mortality rates compared to the sons of non-POW Civil War veterans. These data were taken as an indication that aberrant phenotypes of progeny were a result of their parents experiencing stress from being in forced captivity [3]. Importantly, we must emphasize that the evidence for non-genetic inheritance in humans is strictly epidemiological—ecological and cultural factors cannot be ruled out. Thus, for a number of reasons reviewed elsewhere, epigenetic inheritance in humans has yet to be convincingly demonstrated [4].

**Figure 1:**
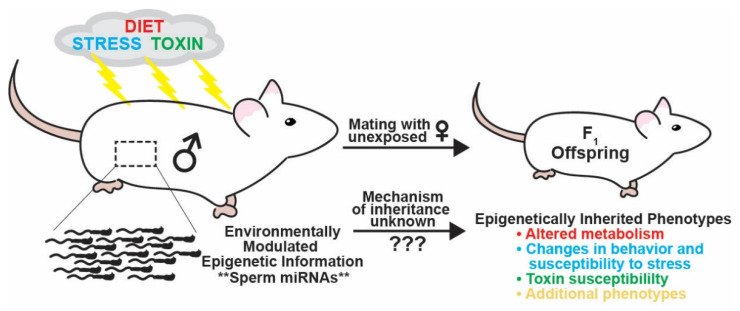
Schematic illustrating how different paternal environmental exposures can influence inherited phenotypes of sired offspring. Environmental perturbations experienced by fathers such as changes in diet, stress, and chemical exposure can transmit non-genetically inherited phenotypes to offspring including altered metabolism, behavioral changes, and modulated susceptibility to stress and chemical exposure. The transmission of paternal environmentally modulated inherited phenotypes is thought to occur by changes in epigenetic information in sperm. Inherited information is encoded in small regulatory RNAs in the best-characterized paradigms of inter- and trans-generational epigenetic inheritance, RNA interference in *C. elegans*, and paramutation in plants. Accordingly, miRNAs have been identified as potential, and in some instances confirmed, carriers of paternal environmentally modulated epigenetic information transmitting inherited phenotypes to succeeding generations of progeny. However, the mechanisms underlying how an RNA in sperm transferred to the zygote upon fertilization can alter embryonic gene expression and development to generate an inherited phenotype are currently unknown.

While the evidence for epigenetic inheritance in humans is limited, studies using rodents, zebrafish, and non-vertebrates such as *C. elegans* (worms) and *D. melanogaster* (flies) have established that parental experiences of environmental perturbation can be transmitted by both fathers and mothers to their progeny (Figure 1). The study of epigenetic inheritance via the maternal gamete is complicated by the direct impacts of the maternal environment on the fetus (F_1_) during gestation [5]. Further, because F_1_ generation female animals complete gametogenesis during gestation, the gametes that will make the F_2_ generation are also directly exposed to the altered environment. Therefore, in female mammals, the F_2_ and F_3_ generations of exposed females must be studied to demonstrate the non-genetic intergenerational and transgenerational effects, respectively. Additionally, to eliminate the confounding effects of maternal care, F_2_ and F_3_ animals must be raised by unexposed animals [6]. An alternative method to study intergenerational inheritance through the female germline is to isolate eggs exposed to the altered environmental condition, use IVF to fertilize the egg with naive sperm, and implant the embryo into a surrogate naive female.

Perhaps because it is substantially less complicated to study the paternal transmission of environmentally modulated epigenetic information to progeny, there have been significantly more studies demonstrating paternal transmission. Further simplifying the study of the paternal contribution, only functional sperm is required for males to reproduce successfully. The male animal itself and even the act of mating can be eliminated as variables by using in vitro fertilization (IVF). Due to the complications associated with demonstrating true epigenetic inheritance via maternal transmission, we focus this review on paternal transmission. While numerous studies have identified changes in offspring phenotypes as a result of paternal environmental perturbations, very few have conclusively identified the molecule which encodes the transmitted epigenetic information, leaving the field with many open questions.

The three most well-defined carriers of epigenetic information are covalent modifications of DNA (most notably DNA methylation), histone post-translational modifications (PTMs) which regulate chromatin state, and RNAs (including non-coding and small regulatory RNAs). Heritable DNA methylation is critical to development as it is required to properly transmit imprinting from one generation to the next [7]; however, de novo establishment and the maintenance of DNA methylation patterns outside of genomically imprinted regions for the purpose of transferring the experience of parents to progeny is not well established. Additionally, mammalian germ cells and preimplantation embryos undergo extensive reprogramming of DNA methylation, thereby resetting the epigenome every generation. Nonetheless, specific regions, such as imprinting control regions, of both the sperm and egg genomes are resistant to these erasures [7]; thus, DNA methylation as a carrier of parentally transmitted epigenetic information cannot be totally excluded [8]. Indeed, DNA methylation is regulated by the paternal environment, as shown particularly in chemical exposure paradigms [9,10]. However, experiments to causally demonstrate specific DNA methylation sites as a carrier of paternal epigenetic information are difficult to perform and are yet to be utilized in this context.

Histone modifications have been demonstrated to transmit epigenetic information mitotically to maintain cell fate through cell cycles in species ranging from yeast to mammals [11,12]. However, paternal chromatin undergoes drastic remodeling during the later stages of spermatogenesis, as defined by the exchange of histones with small basic proteins called protamines. This protein exchange happens in tandem with the remodeling of the nucleus to generate an extremely compact environment. Only ~1% of histones in mice and ~10% of histones in humans are retained in mature sperm [13,14,15]. While the dramatic loss of histones in mature sperm greatly reduces the amount of information that can be encoded in sperm via histone PTMs, the possibility that information in the male germline can be regulated by the environment and subsequently transmitted during fertilization to modulate offspring phenotypes remains. For example, genetically eliminating specific histone PTMs using mutants for chromatin modifying proteins in the male germline has been shown to induce phenotypes in succeeding generations [16,17,18]. Interestingly, environmental interventions such as nutrient supplementation have been demonstrated to modify these epigenetically inherited phenotypes [19]. However, like DNA methylation, histone PTMs at specific loci in the genome of sperm have yet to be demonstrated causally to transmit phenotypes to succeeding generations.

**Figure 2:**
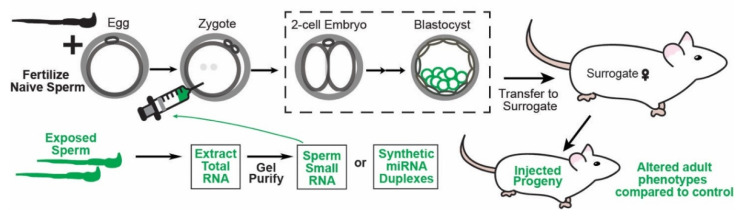
Schematic representing how microinjection of RNA into zygotes can experimentally confirm causal function for RNAs, specifically miRNAs, in transmitting epigenetically inherited phenotypes to offspring in mice. First, zygotes are produced by fertilization either naturally (and then flushed from the inseminated mother) or via IVF with sperm and eggs from naive/control animals and then cultured in vitro. In parallel, RNA from environmentally exposed sperm can be purified and further fractionated to isolate specifically miRNA-size (20–24 nt) sperm RNAs. Alternatively, candidate miRNAs of interest altered in the sperm of animals exposed to the environmental condition of interest can be synthetically produced. Purified sperm RNAs or synthetic RNAs are then injected into the zygotes produced. As the control, zygotes are injected with control sperm RNA, scrambled synthetic miRNAs, or other RNAs for comparisons with the experimentally injected zygotes. The resulting embryos can be analyzed for changes in gene expression and for embryonic phenotypes or transferred to surrogate mothers for full-term development. Finally, fully developed animals are analyzed for the phenotypes transmitted naturally by the paternal environmental exposure of interest.

Consequently, the final carrier of epigenetic information, non-coding RNAs, has received increasingly more attention as a primary candidate for transmitting paternal epigenetic inheritance [20]. Rodent studies over the last decade have found that the paternal environment can modulate the levels of non-coding RNAs, particularly small regulatory RNAs, in the sperm of mammals. These studies have used a variety of stress paradigms, changes in diet, and chemical exposures to demonstrate paternal environmental influences on offspring phenotypes including behavioral changes, susceptibility to stress, altered metabolism, and susceptibility to chemical exposure (Figure 1). While many of these studies identify changes in sperm RNAs due to the perturbation of the paternal environment, only a few have demonstrated causation for the transmission of epigenetically inherited phenotypes. This causation can be confirmed experimentally by demonstrating that the microinjection of the RNA(s) of interest into zygotes naive to the environmental condition results in progeny that exhibit the phenotypes associated with the environmental exposure of interest (Figure 2).

Two classes of small non-coding RNAs have been implicated as carriers of epigenetic information that transfer information through sperm, microRNAs (miRNAs or miRs), and transfer RNA fragments (tRFs or tsRNAs). The composition of sperm small RNAs is unique in that rather than being predominately comprised of microRNAs like most somatic cells, ~80% of their composition are fragments of transfer RNAs and ribosomal RNAs (rRFs or rsRNAs) [21,22]. While originally disregarded as remnants of degradation after spermatogenesis, they have become increasingly recognized as a class of small regulatory RNAs with the ability to contribute non-genetically inherited information intergenerationally. For example, mice fed altered diets exhibit regulated levels of specific tRFs in their sperm (for example tRF-Gly-GCC) and produce progeny with metabolic phenotypes different from their control counterparts [23,24]. Interestingly, this inheritance can be recapitulated in animals developed from naive zygotes microinjected with tRFs purified from altered diet sperm [24]. These findings demonstrate that tRFs can function as carriers of epigenetically inherited information from sperm in mammals. However, much is still to be learned about how tRFs regulate gene expression and whether different species of tRFs from different tRNA isoacceptors have gene regulatory functions.

While the biology of sperm tRFs and their functions in non-genetic inheritance is interesting, a reoccurring theme of many studies involving paternal epigenetic inheritance is that sperm microRNAs can transmit a variety of phenotypes to offspring. However, as with germline DNA methylation and histone modifications, sperm RNAs also have a physiological barrier to overcome to transmit paternal epigenetic information. Sperm in various sexually reproducing organisms are typically hundreds to thousands of times smaller in volume than the eggs they fertilize; thus, the contents of sperm are significantly diluted after fertilization [25]. This introduces a stoichiometric problem: Are there enough sperm RNAs in the zygote to be functional? As more examples of sperm RNAs capable of transmitting paternal epigenetic information to offspring are revealed, mechanisms, which are currently absent, must be described which can account for this stoichiometric problem.

In this review, we focus on the functions of microRNAs in paternal epigenetic inheritance. First, we review the molecular functions of miRNAs in the male germline and sperm, followed by examples of environmentally modulated sperm miRNAs transmitting phenotypes to their progeny. Finally, we speculate on how a sperm miRNA can, after fertilization, alter embryonic development to produce an epigenetically inherited phenotype.

## 2. The Biogenesis and Functions of miRNAs

miRNAs are ~22-nucleotide (nt) small regulatory RNAs that regulate the expression of messengers (mRNAs) via complementary base-pairing. The canonical miRNA biogenesis pathway begins with the transcription of miRNA genes by RNA polymerase II to generate a primary miRNA transcript. This transcript forms a stem-loop structure that is recognized and processed by an endonuclease called the microprocessor complex which consists of Drosha (an RNase III enzyme) and Pasha/DGCR8. Endonucleolytic cutting by Drosha results in a two nt 3′ overhang on the precursor miRNA. This cutting releases a double-stranded hairpin RNA from the rest of the transcript. The hairpin is then exported from the nucleus by an Exportin5/RanGTP complex. In the cytoplasm, Dicer, another endonuclease RNase III enzyme, cleaves the stem-loop, resulting in the final mature miRNA, a ~22 nt double-stranded duplex. One strand of the duplex is loaded into Argonaute (AGO) proteins and serves as a guide for the miRNA-induced silencing complex (miRISC) to repress target mRNA stability and/or translation [26]. Both the 5p and 3p strands (arising from the 5′ or 3′ ends of the pre-miRNA hairpin, respectively) can be loaded into AGO. Typically, the strand with the lower 5′ end stability is loaded into AGO and becomes the guide strand [27]. The remaining strand, called the passenger strand, is degraded.

miRISC mediates gene silencing via complementary base-paring interactions with mRNA transcripts. Most animal miRNAs target mRNA transcripts via complementary base-pairing of the 3′ untranslated region (UTR) of mRNA and nucleotides two to seven of the miRNA, known as the “seed sequence”. Because the seed sequence is the only region required to have perfect complementarity, each miRNA can potentially regulate hundreds of mRNA transcripts. Seed complementarity between miRNAs and their targets facilitates the computational prediction of both mRNA targets of a miRNA and the identification of specific miRNAs that target a given mRNA transcript. Multiple algorithms that predict miRNA and mRNA interactions rely on sequence alignments, the number and strength of predicted seed target sites, and the conservation of target sequences between species to increase the accuracy of predictions [28]. While miRNA target predictions are often used to identify mRNA targets of a miRNA or to identify miRNAs that target a given mRNA, they remain imperfect, often producing both false positive and negative results. When compared to experimental data determining the direct targets of miRNAs, such as by AGO RNA immunoprecipitation (RIP, with additionally modified protocols such as CLIP and CLASH), there are often highly ranked predicted targets that remain experimentally unconfirmed, as well as experimentally validated targets that computational algorithms fail to predict [29]. Therefore, while miRNA target prediction can be a powerful tool for identifying potential targets of miRNAs and generating hypotheses, direct targeting should be confirmed experimentally. Nonetheless, computational prediction algorithms will continue to improve alongside the accumulation of studies further uncovering the biochemical basis of miRNA targeting [30]. The inaccuracies of current computational algorithms may also be due to the variable extents of miRNA and mRNA complementarity which cause the differential modulation of gene silencing (decreasing mRNA stability, repressing translation, or AGO-dependent mRNA slicing). Commonly in plants and very rarely in animals, miRNAs bind target mRNAs with full complementarity, leading to miRNA and mRNA degradation through endonucleolytic cutting called “slicing”. While base-pairing outside of the seed sequence can enhance binding and target preference, typically, seed nucleotides are sufficient for regulation. Non-canonical target complementarity has also been identified including “centered site” base-pairing and seed mismatches with the compensatory pairing of the 3′ end of the miRNA [31,32].

## 3. Sperm microRNAs

Numerous studies have demonstrated that miRNAs are required to produce functional spermatozoa and are essential for male fertility in mice via *Cre*/*LoxP* genetics. To fully review the functions of sperm miRNAs after fertilization in the developing progeny, we first highlight some of the functions of miRNAs during sperm development. The essential nature of miRNAs has been demonstrated by the conditional elimination of *Dicer* or *Drosha* specifically in the male germline. These conditional mutants are infertile due to disrupted spermatogenesis characterized by the depletion of spermatocytes and spermatids leading to significantly depleted mature sperm levels [33]. Multiple stages of spermatogenesis require the functions of specific miRNAs. For example, *miR-221* and *miR-222* are required to maintain undifferentiated pools of spermatogonia [34], while the *miR-34*/*449* family are required later for progression through spermatogenesis [35]. Hundreds of miRNAs are expressed during sperm development, some constitutively and others during specific stages of the process [36]. Additionally, miRNAs are essential for the proper functions of Sertoli cells. These somatic cells, located in the seminiferous tubules of the testis, aid spermatogenesis by nourishing germ cells throughout their development [37]. Specific miRNAs are expressed in Sertoli cells and regulated by androgens [38]. *Dicer* ablation in Sertoli cells using *Amh-Cre* disrupts spermatogenesis and leads to testicular degeneration and infertility, indicating that miRNA functions in somatic Sertoli cells are required for proper sperm development [39,40].

For the purpose of this review, functionally relevant miRNAs were present in mature sperm and subsequently transferred to the zygote during fertilization. Notably, sperm small RNA levels, including miRNAs, are highly dynamic even after the completion of meiosis and differentiation in the testis. After the initial development of sperm in the testis, sperm enter the epididymis, a long, convoluted tubule in which spermatozoa complete maturation and are stored prior to ejaculation. Transit through the epididymis spans several days in humans and two weeks in mice [41]. Sperm morphology does not change significantly during epididymal transit, but sperm do undergo extensive molecular transitions by acquiring molecules required for motility and capacitation. For example, sperm acquire proteins required for motility by fusing with extracellular vesicles (EVs) called epididymosomes that are released from the epididymal epithelium [42].

More recently, several distinct experimental findings established epididymosomes as remodelers of the sperm small RNA payload: (1) The small RNAs present in transcriptionally quiescent mature sperm in the testis are predominately Piwi-interacting RNAs (piRNAs), while epididymal sperm small RNAs are primarily tRNA-fragments (tRFs) and miRNAs [23]. (2) The small RNA composition of mature sperm closely matches the small RNA composition of purified epididymosomes [23,43,44]. (3) In vitro fusion assays demonstrate that the incubation of testicular and caput (early epididymis) sperm with caput or cauda (late epididymis) epididymosomes can transform the small RNA profile of both to more closely resemble the small RNA profiles of caput or cauda sperm, respectively [23,43,45,46]. (4) Paternal epigenetic inheritance can be conferred by naive sperm incubated with environmentally exposed epididymosomes (see below) [47,48]. (5) Metabolically labeled epididymal RNAs expressed in the epididymis can be detected in sperm [45]. (6) Finally, through the transfer of small RNAs via epididymosomes from the epididymis to sperm, the miRNAs present in sperm undergo a dynamic transition. In particular, miRNAs that originate from genomic clusters, including *miR-34b/c*, *miR-17-92*, and *miR-880*, substantially increase as sperm transit into the cauda epididymis [23,43,49]. Together, these findings clearly demonstrate the ability of the epididymis to modulate sperm small RNAs, particularly miRNAs, throughout epididymal transit. Thus, the epididymal influence on the sperm small RNA payload provides a way for epigenetic information to cross the soma-to-germline barrier, often referred to as the Weismann barrier (see Section 10). Contrarily, the degradation or loss of RNAs in sperm during epididymal transit is poorly understood. During maturation in the epididymis, sperm discharge much of their RNAs through the loss of the cytoplasmic droplet [50]. Taken together, the RNAs present in sperm are the result of an equilibrium of RNAs acquired by fusion with epididymosomes, the loss of RNAs through the cytoplasmic droplet, and RNAs retained during the development in the testis.

## 4. Sperm miRNAs Are Important for Embryonic Development

miRNAs make up between 10–20% of the small RNA content (<40 nt length RNAs) of mouse sperm, with tRFs, piRNAs, and ribosomal RNA fragments making up the remaining composition [23,51,52]. Despite their overall depletion relative to miRNA-rich somatic cells, sperm miRNAs have been demonstrated, by at least two studies, to be essential for early embryonic development. Using conditional *Cre*/*Lox* genetics, miRNAs have been depleted in sperm using *LoxP* alleles of *Drosha* and *Dicer* and a germ cell-specific *Stra8::Cre*. The resulting sperm from these conditional mutants were used to fertilize eggs to determine the sperms’ fertilization capacity and their ability to support normal embryonic development. While both alleles interfere with miRNA biogenesis, thereby depleting miRNAs, *Dicer* mutations also ablate the production of endogenous small interfering RNA (endo-siRNAs). Thus, the causality of any observed phenotypes is difficult to assign to specific types of small RNAs. Both male germ-cell genetic disruptions were infertile using standard mating; however, using intracytoplasmic sperm injection (ICSI), *Dicer* and *Drosha* male germ cell conditional knockouts (cKO) with altered miRNA profiles were shown to successfully fertilize WT eggs. Nonetheless, the resulting embryos exhibited reduced competency from fertilization and throughout the preimplantation embryonic development up to the blastocyst stage. Further, embryos produced by miRNA-deficient sperm had significantly diminished post-implantation developmental competency when transferred to surrogate mothers for full-term development. These phenotypes could be rescued, at least partially, when injected with either WT sperm total RNA or gel-purified WT sperm small RNAs [53]. Interestingly, *Dicer* mutant sperm embryo phenotypes were rescued more efficiently with WT sperm total RNA injections while *Drosha* mutant sperm-fertilized embryo phenotypes were rescued more efficiently with WT sperm small RNA injections. These findings demonstrated, for the first time, that sperm-delivered miRNAs are important for normal embryonic development in mice. Additionally, the gene expression for at least a subset of mRNAs, which were computationally predicted to be targets of the dysregulated embryonic miRNAs, were altered in embryos fertilized by *Dicer* and *Drosha* cKO mutant sperm [53]. Thus, as predicted for the function of miRNAs to post-transcriptionally regulate gene expression, the embryonic phenotypes associated with miRNA-depleted sperm are correlated with abnormal embryonic gene expression.

The naturally occurring phenomenon of miRNA transfer from the epididymis to sperm is also required for proper embryonic development in mice. Using ICSI, embryos fertilized by caput sperm exhibited an upregulation of transcripts produced by ~50 genes compared to cauda fertilized embryos via single-embryo RNA-Seq from the four-cell to the blastocyst stage. These genes include important regulatory factors such as chromatin-modifying enzymes and RNA-binding proteins. Additionally, embryos fertilized by caput sperm exhibited defects in peri-implantation development after transfer to a surrogate for full-term development. Strikingly, both the gene expression and developmental phenotypes associated with caput fertilized embryos were completely rescued when they were injected with small RNAs purified from cauda epididymosomes [49]. Further, the phenotypes associated with caput sperm-fertilized embryos could also be rescued by gel-purified 18–26 nt epididymosomal small RNAs comprised primarily of miRNAs. These findings demonstrate that miRNAs acquired during epididymal transit can regulate early embryonic gene expression and later promote implantation. In a follow-up study, gene expression phenotypes associated with caput embryos were successfully rescued by injecting synthetic RNAs mimicking some of the most abundant sperm miRNAs acquired in the epididymis (see above) [23]. Notably, gene expression was rescued significantly more when double-stranded miRNA-mimicking duplexes were used compared to single-stranded mature miRNA-mimics [54]. This finding aligns with literature demonstrating that the loading of mature miRNAs into AGO is initiated by a double-stranded duplex prior to strand separation and insertion of the single mature strand into the protein [55]. Synthetic single-stranded miRNAs are loaded into AGO with significantly reduced efficiency, a fact that should be considered when designing miRNA microinjection experiments to recapitulate sperm RNA-transmitted phenotypes (Figure 2) [56].

The essentiality of sperm miRNAs acquired during epididymal transit for embryonic development is controversial given that several studies demonstrate that caput-fertilized embryos are fully developmentally competent, a fact addressed by the authors of the study described above in their discussion [57,58,59]. However, this controversy is partially explained by considering the method of sperm preparation used for ICSI. Because only sperm heads are injected to fertilize an egg during ICSI, sperm tails are typically sonicated or detached using the piezo required for mouse ICSI. However, Conine et al. removed sperm tails by passing sperm through a 26-gauge needle, thereby creating a pressure vacuum which dissociates the sperm head and tail. Interestingly, embryos fertilized by caput sperm prepared by sonication are capable of full-term development, while embryos fertilized by needle-sheared caput sperm fail to properly implant [60]. It remains unclear why needle shearing renders caput sperm competent for fertilization and preimplantation development yet defective for implantation, and further, why this phenotype is rescued by the injection of sperm miRNAs [61]. One hypothesis that may explain this phenomenon is that epididymosomes containing sperm miRNAs transiently interact with sperm in the caput epididymis before fusing with sperm during epididymal transit. Transient vesicle and sperm membrane interactions in the caput could be susceptible to disruption by needle shearing [61]. There is some evidence for a miRNA internalization process that occurs in sperm given that sperm miRNAs have been shown to become deeply embedded in the sperm nucleus and retained even after detergent exposure or the complete removal of the exterior cell membrane of cauda sperm [62]. However, the dynamic localization of miRNAs transmitted by epididymosomes to sperm during epididymal transit has not been characterized.

In support of the findings discussed in this section, total sperm RNA has also been demonstrated to be required for embryonic development. Mature mouse sperm treated with lysolecithin, pronase, and RNases A and H removes 90% of the RNA associated with sperm. Using RNase-treated sperm for ICSI produced embryos with significantly reduced preimplantation development, as assessed by decreased blastocyst formation rates and live birth rates [63]. Thus, depleting RNA from sperm affects the development of subsequently fertilized zygotes presenting an additional and parallel demonstration of the requirement for sperm RNAs in early embryonic development in mice.

## 5. The Paternal Environment Regulates miRNAs in Sperm That Control Offspring Phenotype

There are many examples of the paternal environment regulating miRNA levels in sperm which are highlighted in the following section and in Table 1. However, only a handful of studies demonstrate causality between the environmental regulation of sperm miRNAs and the elicited phenotype in offspring. These studies are typically performed by purifying the sperm small RNAs from males exposed to the environmental perturbance and then microinjecting those RNAs into a zygote fertilized by a naive (non-exposed) sperm (Figure 2). Additionally, synthetic miRNA duplexes that mimic the natural sperm miRNAs can be injected to confer the regulation associated with the environmental exposure. These microinjected zygotes can then be profiled phenotypically to see whether they display the phenotypes shown to be transmitted by natural conception via exposure to treated males. Below, we highlight specific examples of both indirect correlative connections between the environment, sperm miRNAs, and offspring phenotypes, as well as examples where sperm miRNAs have been directly demonstrated to transfer epigenetically inherited phenotypes from father to offspring.

## 6. Paramutation

One of the first examples of RNA-mediated inheritance was demonstrated in mammals in a phenomenon called paramutation. Paramutation was first demonstrated in plants as a heritable epigenetic modification that occurs through the interaction of two different alleles at the same genetic locus, whereby the phenotype of one allele is transmitted to progeny in a ratio exceeding that predicted by Mendelian inheritance. Further, the allele corresponding to the dominantly inherited phenotype, referred to as the paramutagenic allele, can direct the conversion of non-paramutagenic alleles, referred to as paramutable alleles, to become paramutagenic in succeeding generations [64]. Mechanistic studies of paramutation in plants have determined that the phenomenon occurs through epigenetic gene silencing by histone modifications and DNA methylation, while trans-interactions between different alleles are conferred by small RNAs [65]. In mice, a null mutant of the *Kit* gene (*Kit^tm1Alf^*), which encodes a tyrosine kinase receptor involved in melanin synthesis, was generated by a *lacZ* insertion downstream of the start site of *Kit* and was shown to exhibit paramutation. Heterozygous *Kit^tm1Alf^*/+ male and *Kit^tm1Alf^*/+ female mating crosses produced WT (+/+) progeny that displayed *Kit^tm1Alf^*/+ phenotypes characterized by white feet and a white tip of the tail, indicating that *Kit^tmAlf^* is paramutagenic and capable of converting WT alleles to a silenced state [66]. Further, genotypically WT offspring exhibited altered levels of *Kit* mRNAs, with decreased levels of full-length poly-adenylated *Kit* mRNA transcripts and an accumulation of small RNA fragments compared to naive WT animals. No significant changes in either cytosine or histone methylation were observed between WT, heterozygous, or paramutated animals (+/+ genotype from a parent carrying a *Kit^tm1Alf^* allele) [66]. In investigating how silencing was inherited, *Kit^tm1Alf^*/+ and +/+ paramutated animals were found to carry elevated levels of *Kit* RNA fragments in their sperm, particularly in later spermatogenic stages. Astoundingly, the microinjection of total RNA from *Kit* heterozygotes, isolated from either the sperm or brain, into naive WT (+/+) zygotes produced progeny with the paramutated phenotype (white tail tips). Further, the microinjection of *miR-221* and *mir-222*, which target *Kit*, into naive WT zygotes also produced mice with white tail tips. This implies that regulation of *Kit* by microRNAs in early development can also produce epigenetically inherited, or paramutagenic, phenotypes [66]. Two caveats of this study should be noted: First, white tail tips were occasionally observed in animals developed from naive WT zygotes injected with control RNAs (either *lacZ* RNA or total RNA isolated from naive WT animals). However, this phenotype was observed significantly less frequently compared to when RNA from isolated paramutated mice or miRNAs were used for injections. Secondly, single-stranded *miR-221* and *miR-222* zygotic microinjections were performed to confer the paramutated phenotype. Double-stranded miRNA duplex injections were not performed. Thus, the functions of *miR-221* and *miR-222* that confer paramutation may not be through canonical regulation by miRNAs and AGO (see above). Rather, these effects may occur through an alternative mechanism initiated by sequence homology to *Kit* mRNA.

Other paramutation-like phenomena have been demonstrated in mice with the similarly remarkable ability to be transmitted by sperm miRNAs. For example, microinjection of either twenty nt fragments homologous to the coding sequence of the cell-cycle regulator *Cdk9* or sense *miR-1*, which targets *Cdk9*, into zygotes were shown to produce animals with cardiac hypertrophy [67]. When gene expression in the developing heart of *miR-1* zygotic injection animals was assayed, *Cdk9* expression increased two-fold and *miR-1* RNA levels remained unchanged compared to control-injected mice [67]. The upregulation of the *miR-1* targets *Cdk9*, rather than the downregulation expected by the canonical function of miRNAs, paired with the finding that sense *Cdk9* RNA fragments induce similar phenotypes suggest that an unknown regulatory mechanism occurs in this paradigm to produce phenotypes in the developing heart.

In a similarly conducted study, the injection of *miR-124* sense RNA into zygotes produced mice that are 30% larger than control-injected animals, thus named the “giant phenotype” [68]. This phenotype manifested as early as the blastocyst stage, where *miR-124*-injected embryos frequently had duplications of the inner cell mass resulting in twin pregnancies [68]. Interestingly, *miR-124* zygotically-injected male mice (referred to as miR-124*) produced similarly giant offspring when mated to control WT females. This phenomenon was also demonstrated when miR-124* female mice were crossed to WT males, indicating that the epigenetically inherited phenotype can be transmitted by both germlines. The inheritance of the paramutated giant phenotype could be propagated for two generations in crosses initiated by both male and female miR-124* mice, with F_3_ generation progeny returning to normal size [68]. Further, elevated levels of *miR-124* RNAs were found in the testis of miR-124* males indicating that the transgenerational regulation of *miR-124* could potentially be transmitting the giant phenotype between generations. Fascinatingly, mice with transgenic sperm-specific overexpression of *miR-124* RNA driven by a strong spermiogenic promoter (*Prm1*) produce progeny with the giant phenotype when mated with WT females [68]. As with the example of *miR-1*, predicted targets of *miR-124* (*Sox9*, *LamC1*, and *Acaa2*) were upregulated in miR-124* embryos compared to controls. These findings again suggest that the inheritance of the paramutated phenotype is likely produced by an unknown non-canonical miRNA regulatory mechanism. In search of this regulatory mechanism induced by zygotic injection of *miR-124*, increased PTMs including H3K9me2 and H3K9me3 were found at a putative upstream regulatory element of *Sox9* compared to controls. However, because these marks are typically associated with gene silencing and heterochromatin formation, it remains difficult to explain the upregulation of *Sox9* in miR-124* embryos. Nonetheless, these data establish that *miR-124* injections can influence the chromatin of targeted genes (see Discussion).

While the published examples of the phenomenon of paramutation in mice that are described here can all be induced by the injection of miRNAs into zygotes, the epigenetically inherited phenotypes are likely due to non-canonical miRNA-dependent regulatory mechanisms. In all instances, the single-sense strand of the miRNA rather than duplex miRNA was injected, suggesting that the functions these injected miRNAs are initiating are possibly through a non-canonical miRNA pathway (see below). Additionally, the predicted mRNA targets of the injected single-stranded miRNAs are all upregulated relative to controls, whereas mRNA downregulation is expected if the injected miRNAs were functioning in a canonical manner. While the examples of zygotic injection of miRNAs inducing paramutated phenotypes in mice are likely the result of a novel regulatory mechanism, they are nevertheless interesting in the context of this review as they are initiated by the microinjection of RNAs shortly after fertilization or are directly delivered by sperm (Figure 2). These results provide a unique demonstration of the ability of sperm RNAs to regulate embryonic development and offspring phenotype.

## 7. Diet and Metabolism

Many studies have linked changes in a paternal diet with altered phenotypes in progeny, specifically regarding changes in offspring metabolism. Using rats as a model, animals fed a high-fat diet (HFD) for 12 weeks produce F_1_ progeny of both genders with decreased body weight and increased glucose tolerance when fed a control diet. Interestingly, the female progeny of HFD fathers were resistant to HFD-induced weight gain, while male progeny did not demonstrate any major phenotypic differences compared to controls [69]. Interestingly, the miRNA *let-7c*, which influences glucose metabolism and insulin sensitivity, was shown to be upregulated, while *miR-293-5p* and *miR-880-3p* were downregulated in the sperm of both HFD-fed fathers and control diet-fed F_1_ male progeny compared to controls. In agreement with the observed changes in the sperm miRNA content of F_1_ males, F_2_ females sired by these males which were fed a control diet also exhibited aberrant metabolic phenotypes including decreased birth weight, increased glucose tolerance, resistance to HFD-induced weight gain, and defective glucose tolerance when exposed to HFD. F_2_ male siblings of these animals which were fed a control diet only developed increased insulin levels during glucose tolerance tests and displayed no other aberrant phenotypes. Further, *let-7c* was also differentially expressed in the metabolic tissues of both F_1_ and F_2_ offspring of HFD fed fathers only when fed HFD (Table 1). Although the authors hypothesized that *let-7c* may reprogram sperm to produce an HFD-induced phenotype in offspring, no causality was demonstrated for the miRNA in transmitting epigenetically inherited phenotypes modulated by paternal diet.

Similarly, male mice fed an HFD to induce obesity also produced F_1_ offspring of both genders which displayed increased body weight and insulin resistance compared to control progeny [70]. Interestingly, the F_2_ progeny sired by these F_1_ males did not exhibit any measurable metabolic phenotypes, while female F_2_ progeny displayed insulin resistance and increased adiposity compared to controls, indicating that the diet-induced phenotype could be transmitted transgenerationally, to some extent [70]. Four miRNAs were dysregulated in sperm of F_0_ founder males fed HFD compared to the control. While unexposed F_1_ males also sired female offspring with altered metabolic phenotypes resembling that of the HFD-fed male progeny, a distinct subset of miRNAs was differentially regulated in F_1_ sperm. In sperm from F_1_ males, eleven miRNAs were upregulated and two were downregulated compared to sperm from control animals [71] (Table 1). While these results are inconsistent with the same miRNAs transmitting metabolic phenotypes from F_0_ to F_1_ to F_2_, the authors hypothesize that perhaps a different subset of miRNAs or other forms of epigenetic information may be transmitting information intergenerationally (F_0_ to F_1_) and transgenerationally (from unexposed F_1_ to F_2_). Nonetheless, causality for the sperm miRNAs as the carrier of the inherited phenotype regulated by HFD was not demonstrated during these experiments, leaving the true carrier of epigenetic information in this paradigm unknown.

More nuanced dietary changes can also induce heritable phenotypes in succeeding generations through the male lineage. For example, HFD has been administered with either lard-based HFD (high saturated fatty acids) or corn oil-based HFD (high n-6 polyunsaturated fatty acids) to male rats. Female offspring of lard-fed male rats raised on a control diet presented with increased birth weight and weight gain, while female offspring of corn oil-fed male rats had higher fasting glucose levels and less weight gain compared to their lard-fed counterparts yet more than the control group [72]. Interestingly, these offspring also had differential responses to carcinogen (DMBA) administration that induces mammary tumor formation. The lard diet group exhibited increased mammary tumor incidence with fewer apoptotic cells, an increased number of proliferative cells, and a decreased number of apoptotic cells in mammary gland lobules. However, the female progeny of oil-fed fathers had decreased tumor latency, growth, and multiplicity compared to their lard counterparts. Mammary cells exhibited decreased levels of apoptosis in mammary tumors. Based on microarray data, corn oil-fed male rats had 89 downregulated miRNAs in their sperm compared to lard-fed male rats. Furthermore, female offspring of corn oil-fed male rats had 21 downregulated and 2 upregulated miRNAs in their mammary glands compared to female offspring of lard-fed male rats. Three miRNAs, predicted to regulate signaling pathways associated with growth hormone, phosphatase and tensin homolog (PTEN), prolactin signaling, and cardiac hypertrophy, were downregulated in both sperm of oil-fed male rats and the mammary glands of their female progeny (Table 1).

Similarly, a mouse model demonstrated that diet-induced paternal obesity at the time of conception induces changes in sperm miRNAs and that progeny sired by these animals exhibited phenotypes such as changes in birth weight, delayed mammary gland development, and higher rates of carcinogen-induced mammary tumors [73]. As in the rat model, a subset of miRNAs was downregulated in obese father’s sperm and their progeny’s mammary tissue. Further, hypoxia signaling, which is regulated by *miR-874*, was coordinately downregulated in mammary tissue from the progeny of obese fathers [73] (Table 1). However, it should again be noted that neither model (mouse nor rat) demonstrated causality for sperm miRNAs in transmitting diet-induced epigenetically inherited phenotypes. Further, while the same miRNAs dysregulated by dietary exposure in both exposed sperm and the mammary tissue of female offspring in these studies, the mechanism for how this phenomenon may occur remains unclear. The results of these studies imply that the change in sperm miRNA levels is somehow perpetuated after fertilization and throughout development specifically in the mammary tissue. A self-regulating feedback mechanism of these specific miRNAs, in a manner that is maintained and lost in specific tissues throughout development, would be a provocative mechanism to explain such a phenomenon.

There is at least one example causally demonstrating that changes in miRNAs in sperm or present during fertilization can directly transmit a paternal diet-induced epigenetically inherited metabolic phenotype in offspring. Mice fed a Western-like diet (WLD) high in both fat and sugar were mated to control females and their progeny were raised on a control diet. Both male and female progeny presented with increased body weights, while male progeny specifically had altered fasting blood glucose levels and hyperglycemia in response to glucose and insulin injections compared to control offspring [74]. Importantly, injecting sperm RNAs from mice fed WLD into naive zygotes was shown to produce fully developed mice with phenotypes resembling those of the progeny of WLD fathers. Zygotes injected with sperm RNAs isolated from control-fed males did not exhibit any measured phenotypes. In search of the RNAs responsible for the transmission of the phenotypes, four miRNAs were found to be dysregulated in both the testis and sperm of WLD-fed males compared to control diet-fed males. While *miR-19b* and *miR-29a* were the two most abundant dysregulated miRNAs, microinjection of single-stranded *miR-19b* alone produced mice with increased body weight. These zygotically RNA-injected mice exhibited no increase in fasting glucose levels and variable glucose tolerance and insulin sensitivity, suggesting that the sequelae of phenotypes inherited from WLD are transmitted by different miRNAs or other epigenetic carriers. Interestingly, when male mice that were zygotically injected with *miR-19b* were mated to control female mice, some of the resulting offspring developed the full metabolic phenotype despite their sires being fed a normal diet [74].

Another recent study using WLD mice demonstrated that five successive paternal generations fed high-fat and -sugar diets exhibited an increasingly exacerbated overweight phenotype and accelerated obesity-associated pathology development. A separate subset of both male and female F_1_ offspring fed a control diet displayed impaired glucose tolerance and were heavier than their control counterparts [75]. Although miRNAs were not specifically analyzed, microinjections of total RNA from the sperm of either the first or fifth consecutive sire fed WLD into naive zygotes produced male progeny that presented with increased body weight and altered glucose and insulin responses. When mating these males to control females, metabolic phenotypes were partially transmitted to F_2_ offspring when the sperm RNAs from the first generation of WLD fed mice were used. Interestingly, aberrant phenotypes were transferred through the F_3_ generation progeny when embryos were injected with RNA from the fifth WLD consecutive sire. These results imply that the accumulating effects of consecutive generations being fed WLD can transfer more stable germline epigenetic modifications that persist to at least the following four generations of control diet-fed offspring. However, when only one generation is fed WLD, transgenerational features may be induced to a weaker extent and to fewer subsequent generations.

Paternal exercise has also been shown to affect metabolic and behavioral phenotypes in offspring. When male mice were exposed to twelve weeks of voluntary wheel-running, their resulting progeny were more susceptible to the adverse effects of HFD as demonstrated by increased body weight, adiposity, impaired glucose tolerance, and elevated insulin levels. The sperm of runners exhibited significantly differentially expressed levels of four miRNAs (Table 1) [76]. In another example, when male mice were subjected to voluntary running for four weeks, the progeny of male runners had suppressed reinstatement of juvenile fear memory and reduced anxiolytic behavioral phenotypes. Female offspring did not display any of these phenotypes. Three miRNA species were found to be altered in the sperm of runners (Table 1) [77]. However, the causality of the miRNA in the transmission of paternal exercise-induced phenotypes in progeny was not demonstrated in either of these examples.

## 8. Stress

Numerous types of stress exposures, including but not limited to physical, social, and mental stresses have been linked to adverse physiological responses including in the sympathetic nervous system, immune system, and the hypothalamic–pituitary–adrenal (HPA) axis which is responsible for corticosterone stress hormone production and regulation. This stress-induced dysregulation can produce a variety of conditions including psychiatric disorders, cardiovascular disease, altered metabolism, and inflammation [78]. Further, parental stress to both the mother and father has been associated with the non-genetic inheritance of psychiatric conditions and the transmission of stress-related vulnerabilities in progeny [79,80]. Many studies demonstrate that paternal stress produces non-genetically inherited phenotypes in offspring, with several of these studies identifying small non-coding RNAs, including miRNAs, as potential mediators of stress-associated phenotypes.

One of the first examples of sperm RNAs transmitting environmentally modulated epigenetically inherited phenotypes to offspring was an experimental model using male mice exposed to traumatic stress in early life via unpredictable maternal separation combined with unpredictable maternal stress (MSUS). These males, when mated to control females, produced male offspring with reduced avoidance and fear, altered response to aversive conditions, and altered behavioral despair as shown by an elevated plus maze, a light-dark box, and a Porsolt forced swim test, respectively. In addition, F_1_ offspring of MSUS fathers presented with metabolic phenotypes in the form of decreased insulin and glucose levels compared to control progeny. All the phenotypes exhibited in F_1_ male progeny were also found in the F_0_ males directly exposed to the stress. Furthermore, MSUS males had elevated levels of *miR-375-3p*, *miR-375-5p*, *miR-200b-3p*, *miR-672-5p*, and *miR-466-5p* in their sperm, serum, hippocampus, and hypothalamus, indicating that the stress similarly affects miRNAs in the brain and germline (Table 1) [81]. While the F_1_ offspring did not have the same miRNAs altered in their sperm, the five miRNAs elevated in their father’s sperm were altered in their serum and hippocampus. It appears that there could be functional effects of stress-induced changes in miRNAs, as demonstrated by the downregulation of *Catenin β1*, a target of *miR-375*, in the hippocampus in F_1_ offspring, indicating that stress in the previous generations can potentially produce molecular changes in the brains of the succeeding generation. Interestingly, the F_1_ generation, which was not directly exposed to MSUS, had more exacerbated phenotypes compared to their fathers who were directly exposed to traumatic stress in early life. Subsequently, when unexposed F_1_ males (sons of MSUS males) were mated to control females, their male progeny also presented with behavioral phenotypes like F_0_ and F_1_ animals. However, similarly to the transgenerational transmission of diet (described above), F_1_ males did not exhibit the same changes in their sperm miRNAs as MSUS exposed males [81].

To determine the carriers of epigenetic information transmitting the phenotypes inherited by the progeny of MSUS males, total RNA from F_0_ sperm of MSUS treated or untreated mice was purified and microinjected into naive zygotes fertilized by unexposed sperm, resulting in animals with the same behavioral and metabolic phenotypes as natural MSUS offspring. Offspring sired by mice that were injected with stressed sperm RNA as zygotes also displayed depressive-like behaviors indicating possible transmission of information from injected sperm RNAs in the previous generation. In addition to recapitulating the behavioral and metabolic phenotypes present in MSUS offspring by RNA injection, *miR-375* was upregulated and its target *Catenin β1* downregulated in the hippocampus of zygotically-injected animals [81]. These findings demonstrate the capacity of sperm total RNA, but not specifically miRNAs, in regulating a phenotype in offspring and specifically a molecular phenotype in the brain. Notably, the regulation of *Catenin β1* by *miR-375* is not required for the behavioral phenotypes presented in MSUS progeny, as F_2_ animals presenting with the same phenotypes do not exhibit this miRNA/target regulation in their hippocampus. Given that miRNAs are not altered in the sperm of F_1_ animals nor in brain tissue of the F_2_ generation, the authors suggest that the altered sperm RNAs encode information through other epigenetic marks to transfer information to subsequent generations. However, the direct causality of miRNAs in transmitting the inherited phenotype through the zygotic injection of specific miRNAs or epigenetic alterations secondary to sperm RNAs was not determined.

The first direct demonstration of sperm miRNAs transferring paternal-stress modulated phenotypes to offspring was by Dr. Tracy Bale’s lab in a series of papers utilizing a chronic variable stress (CVS) paradigm. Male mice stressed for six weeks via CVS, either throughout puberty or as adults, exhibited significantly increased expression of nine miRNAs, *miR-193-5p*, *miR-204*, *miR-29c*, *miR-30a*, *miR-30c*, *miR-32*, *miR-375*, *miR-532–3p*, and *miR-698* in their sperm (Table 1) [82]. When mated to control females, the resulting offspring of both genders had significantly blunted corticosterone responses to acute restraint stress. Further, no significant differences were identified in any of the assessed behavioral measures, serotonergic regulation (which is known to regulate the HPA axis) was intact, and no changes in gene expression in the pituitary and adrenal glands were identified. Therefore, the authors conclude that paternal stress did not induce changes in the physiological stress response of offspring, and reprogramming of the HPA axis lies centrally with remarkable specificity. Further, the progeny of stressed animals exhibited broad changes in gene expression in the paraventricular nucleus (PVN) and bed nucleus of the stria terminalis, key stress regulatory brain regions. To determine if the miRNAs with increased expression in the sperm of stressed males encoded the epigenetically inherited phenotypes, synthetic duplex miRNAs mimicking the nine miRNAs were injected into naive zygotes. Astoundingly, microinjection of these specific sperm miRNAs produced animals which recapitulated the HPA stress axis sensitivity and PVN gene expression patterns in the progeny of stressed mice [83]. To determine the functions of these miRNAs after fertilization, candidate target maternal mRNAs were analyzed for changes in expression by RT-qPCR in embryos 24 h post-fertilization and microinjection. Accordingly, mRNAs such as *Sirt1*, *Ube3a*, *Srsf2*, *IL6st*, *Ncl*, *Aars*, *Agfg1*, and *Ralbp1* were reduced 50–75% in microinjected embryos compared to control-injected embryos. Notably, *Ube3a* and *Sirt1* are known to play a role in chromatin remodeling and neurodevelopmental disorders. The authors hypothesize that sperm miRNAs may initiate lasting, programmatic changes that influence neurodevelopment and stress reactivity of the developing embryo [83]. While this could explain the mechanism for how sperm miRNAs can produce an inherited phenotype in offspring, it is difficult to imagine how such changes in maternal mRNA expression in single-cell zygotes could produce phenotypes in specific tissues in the offspring and avoid producing more broad phenotypes.

Recently, the same paradigm was used to show that paternal stress exposures are a result of signaling from the epididymis to the germline. Using ICSI of naive sperm incubated with EVs isolated from in vitro culture of epididymal epithelial cells treated with corticosterone produced offspring with altered neurodevelopment and adult stress reactivity [48]. Interestingly, eight days after corticosterone treatment of the epididymal cell line, the miRNA profiles matured in a pattern similar to the changes seen in stressed sperm compared to control sperm. Thus, EVs from this timepoint were used for incubation with sperm prior to ICSI. This work demonstrates that the transfer of EVs, from the epididymis to sperm, can transmit environmentally regulated epigenetically inherited phenotypes to offspring. While causation for miRNAs in this transmission was not determined, the fact that miRNAs are acquired by sperm during epididymal transit provides a pathway for the environment to modulate the male reproductive process and facilitate the calibration of sperm miRNA content, and thus offspring phenotype.

Paternal stress has also been shown to play a critical role in the development of depression in offspring with sperm miRNAs being implicated in the inheritance. Male mice were exposed to unpredictable chronic mild stress daily for five weeks to induce depression-like phenotypes including despair and anhedonia in addition to elevated corticosterone and associated decreased body weight gain [84]. The stressed mice also had elevated mRNA expression of corticotropin-releasing hormone (CRH) compared to control mice, indicating excessive HPA axis activation. The progeny (F_1_ generation) of the stressed mice (which experienced either chronic mild stress or chronic restraint stress to induce depression-like phenotypes) mated to control female mice exhibited no aberrant phenotypes under baseline conditions. However, when exposed to slight CVS for two weeks or longer, the mice displayed depressive-like phenotypes like their fathers and had increased *CRH* mRNA and plasma corticosterone levels. Progeny of control male mice not exposed to stress did not display depressive-like phenotypes after CVS exposure for two weeks. Notably, the F_2_ generation, made by mating F_1_ males with normal females, did not show any susceptibility to a CVS stimulus, indicating that transgenerational inheritance of stress does not occur in this paradigm. Fascinatingly, injection of total RNA or small RNAs extracted by MirVana RNA isolation (which enriches for < 200 nt RNAs) from stressed sperm (F_0_ animals) into naive zygotes produced offspring with the same depressive-like behavioral alterations. Further, sixteen miRNAs were upregulated, and one miRNA was downregulated in stressed sperm while the F_1_ generation displayed no significant changes in sperm RNAs compared to controls (Table 1) [84]. To determine if these miRNAs were transmitting the stressed induce phenotypes to the succeeding generation, synthetic miRNAs mimicking the 16 upregulated sperm miRNAs were injected into naive zygotes which again recapitulated the inherited phenotypes compared to synthetic scrambled RNA injected controls. Further confirming the role of miRNAs in the transmission of the phenotype, co-injection of miRNA antisense strands to block the sixteen upregulated sperm miRNAs into zygotes fertilized by stressed sperm attenuated the stress-induced inherited phenotypes [84].

To determine the functions of the miRNAs transmitting the paternal stress-induced phenotypes in offspring, mRNA-Seq was performed on day 3.5 blastocysts developed from zygotes injected with purified small RNAs from stressed sperm and control sperm [84]. One hundred and seven upregulated and one hundred and fifty-seven downregulated embryonic genes were identified by RNA-seq in stressed sperm RNA-injected blastocysts compared to control sperm RNA injected blastocysts. Interestingly, one upregulated gene and 77 downregulated genes were predicted targets of miRNAs upregulated in stressed sperm. To determine if miRNAs regulated by stressed sperm could regulate any of these genes directly, embryonic stem cells transfected with synthetic miRNA mimics displayed reduced expression of six genes (*App*, *Tspan7*, *Wnk3*, *Ly6a*, *Grin3a*, and *βCamkII*) also regulated in embryos. Further, a luciferase reporter assay confirmed the direct binding of the corresponding miRNAs to the 3′UTRs of all 6 genes. Embryos injected with small RNAs from stressed sperm were also confirmed to have decreased levels of all genes except *βCamkII* which was increased from the four-cell to the morula stage. While direct targeting of mRNAs by the stress-regulated miRNAs in embryos injected with stressed sperm RNA was not demonstrated, this experiment does confirm that these miRNAs can directly interact with these targets in a relevant cell type. The authors hypothesized that even the small amounts of miRNAs introduced to embryos via sperm can cause changes to gene expression in the embryo, thereby impairing brain maturation and ultimately inducing a vulnerable phenotype for stress-induced depression. Considering that the early embryo does not have a functional nervous system, how sperm RNAs can program early development in a way that specifically alters the developing nervous system remains unclear.

The previously reviewed studies assessed the effects of stress that were induced by various stress-inducing paradigms; however, the direct effects of elevated corticosterone levels and its ability to transmit phenotypes to succeeding generations of progeny has also been studied. Adult male mice (F_0_ generation) were given 25 ug/mL of corticosterone-supplemented water ad libitum for four weeks and then mated to control females to produce the F_1_ offspring [85]. F_0_ males did not present with any changes in behavior but had a significant decrease in the post-stress response of serum corticosterone and a significant reduction in glucocorticoid receptor mRNA in the hippocampus. Female offspring of corticosterone administered males presented with changes in early-life fear conditioning but no behavioral changes in adulthood. Although F_1_ females did not have any changes in the glucocorticoid receptor, they did have altered anxiety-associated gene expression in the brain. Specifically, *Igf2* mRNA was significantly decreased, and *Bdnf* exon IV transcript levels were significantly greater in the hippocampus. Male F_1_ progeny displayed altered anxiety-related behaviors and significantly increased *Igf2* mRNA expression in the hippocampus. F_2_ animals, generated by mating F_1_ male mice (whose fathers were administered corticosterone) to control females, displayed some behavioral phenotypes presented in the previous generation. Notably, no changes in hippocampal *Igf2* levels were observed in the female progeny while male progeny had increased expression. Together, these results indicate that male offspring were more affected by paternal corticosterone treatment than females in both F_1_ and F_2_ progeny. To determine the epigenetic carrier of the phenotypes inherited by the progeny of corticosterone-administered males, 46 sperm miRNAs were found to be altered two-fold or greater in the male mice treated with corticosterone water. The miRNAs in sperm regulated by corticosterone included *miR-444*, *miR-190b*, *miR-192*, *miR-26b*, *miR-350*, *miR-449a*, *mir-467e*, and *miR-98*. A separate cohort of corticosterone-treated mice was used to successfully validate that *miR-98*, *miR-144*, and *miR-190b* were highly expressed in corticosterone sperm compared to control–water-fed mice (Table 1) [85]. *miR-190b*, *miR-26b*, *miR-350*, and *miR-449a* are predicted to target *Bdnf*, and *miR-192*, *miR-449a*, and *miR-98* have predicted targeting sites for *Igf2*. Although *Bdnf* and *Igf2* are well known regulators of anxiety behavior and fear extinction, the propagation of information from sperm miRNAs to the brain of adult mice is unclear.

Human sperm has also been analyzed for changes in miRNA content in response to stress. Sperm miRNAs were found to be modulated in males who experienced a period of elevated stress compared to males who had little variation in stress levels over time [86]. In another instance, sperm miRNAs associated with stress were compared between humans and mice. Human males exposed to stress in early life presented with sperm with reduced levels of *miR-449a* and *miR-34c* which were also decreased in the sperm of mice exposed to chronic social instability (CSI) stress at the onset of adolescence (Table 1) [87]. Male mice exposed to CSI stress produced female offspring with anxiety-like and sociability defects while the male progeny (F_1_ generation) displayed no obvious behavioral defects. Surprisingly, F_2_ generation female offspring sired by these F_1_ males displayed both behavioral defects. Interestingly, *miR-449a/b* and *miR-34b/c* are not present in mouse eggs; thus, they are delivered solely by sperm to the zygote upon fertilization. Pre-implantation (two-cell through morula stage) embryos isolated from mating stressed male mice to control females had significantly decreased expression of both *miR-449a* and *miR-34c* compared to control sperm fertilized embryos [87]. These two miRNAs have the same seed sequence; therefore, they are likely to have highly overlapping targets which include *p53*, *CDK6*, *c-MYC*, *HDAC1*, and *BCL-2*. These target genes are potent regulators of gene expression and cellular physiology with the capability of altering development. One explanation of these findings is that decreased levels of *miR-449a* and *miR-34c* in the sperm of stressed mammals can feedback into the regulation of their own expression during early embryogenesis thus sustaining regulation during early development of both the miRNAs and potentially their targets. This speculative mechanism provides a potential model for sperm miRNAs having a long-lasting influence during development.

## 9. Toxins

Toxins including environmental pollutants released by humans into the environment and consumable toxins including drugs and alcohol have been shown to alter sperm miRNA content. However, to our knowledge, there are no examples that have demonstrated causality of the miRNAs regulated in sperm in transmitting the toxin-associated phenotypes to offspring using microinjection experiments into naive embryos (Figure 2). Accordingly, we only briefly review the findings of toxins regulating miRNAs in sperm and producing epigenetically inherited phenotypes in offspring.

Environmental pollutants such as persistent organic pollutants (POPs) are increasingly concerning to the global ecosystem and have been recently shown to affect the paternal germline. Male rats exposed to POPs in utero (via gavage feeding to pregnant dams) were demonstrated to have altered sperm miRNA content. This exposure-induced alteration in sperm miRNAs could be intervened with by dietary folic acid (FA) supplementation, which partially restored sperm miRNA profiles across subsequent generations [88]. Sperm from F_0_ male rats exposed only to POPs in utero exhibited ten upregulated miRNAs. Subsequent generations of progeny were produced by mating male offspring with control female rats. The F_1_ offspring of POP-exposed males displayed dysregulation of 37 miRNAs in their sperm. Subsequently, F_2_ and F_3_ progeny presented with ten and one altered miRNAs in their sperm, respectively. Importantly, animals directly exposed (F_0_) to POPs in addition to FA supplementation in utero exhibited only one upregulated miRNA in their sperm. F_1_ offspring sire from these males still presented with 12 upregulated miRNAs in their sperm, which while significant, is notably less than the F_1_ offspring of POPs exposed males. Together, these results demonstrate that folic acid supplementation attenuates the effects of POPs on the sperm miRNA content of exposed mice. Although inherited phenotypes of exposed animals and progeny were not studied in this experiment, it would be interesting to determine in this model if the increased number of altered sperm miRNAs translates to alterations in epigenetically inherited phenotypes.

Vinclozolin is a common fungicide shown to produce atrophy of reproductive cells. Exposure to this toxin was demonstrated to induce transgenerationally inherited phenotypes defined by the increased incidence of diseases including spermatogenic cell apoptosis and kidney abnormalities in succeeding generations of progeny [89]. These epigenetically inherited phenotypes were generated by performing daily IP injections of vinclozolin to pregnant rats on days eight through fourteen of gestation. The male offspring exposed to vinclozolin in utero (F_1_ males) were then used to establish a paternal lineage revealing the inter- and trans-generational effects of vinclozolin exposure on the male germline. When the sperm of the F_3_ generation of vinclozolin lineage were analyzed for small non-coding RNAs (sncRNAs) as compared to control mice, 222 sncRNAs were significantly differentially expressed including thirteen upregulated and eight downregulated miRNAs (Table 1). While these studies of paternal exposures demonstrate that chemicals can induce transgenerationally inherited phenotypes correlated with changes in miRNAs in the sperm of males transmitting the phenotypes, much work remains to causally demonstrate paternal epigenetic inheritance initiated by toxins.

Another correlative example found in humans established that the sperm of cigarette smokers have altered levels of sperm miRNAs compared to non-smokers [90]. 28 miRNAs display changes in the sperm of smokers compared to non-smokers with 21 upregulated and seven downregulated. *miR-146b-5p* and *miR-652* are among the miRNAs downregulated in the sperm of smokers with *miR-509-5p* and *miR-519d* upregulated (Table 1). Intriguingly, these upregulated miRNAs have previously been shown to be altered in the testis, seminal plasma, and/or semen of infertile men.

Finally, ethanol is a highly consumed substance in humans across the world with well-known toxic effects in both humans and mice. In mice, alcohol consumption produces non-genetically inherited phenotypes in offspring and can alter the miRNA content of sperm. In one example, male mice were exposed to ethanol inhalation chambers eight hours a day, five days a week, for five weeks. When the sperm of treated animals were analyzed for changes in small RNAs, alcohol-exposed mice displayed increased levels of seven miRNAs (Table 1). Interestingly, the same sperm sncRNAs altered in sperm were also altered in the epididymosomes of exposed mice [91]. When sperm from naive control mice were incubated with epididymosomes from ethanol-treated mice and then used to fertilize control eggs via IVF, the progeny presented with aberrant phenotypes compared to eggs fertilized by sperm incubated with naive control epididymosomes. The male offspring exhibited altered body weights and modestly changed binge ethanol drinking behaviors, while female offspring had basal anxiety-like behaviors and sensitivity to an anxiolytic dose of ethanol [47]. These results provide further evidence that small RNAs, specifically miRNAs, may be altered in sperm through soma (epididymis), rather than by direct regulation in the germline, to impart intergenerational effects. However, the ability of epigenetic information in epididymosomes to produce transgenerational epigenetically inherited phenotypes in succeeding generations of progeny (F_2_ and beyond) has not yet been determined.

## 10. Conclusions and Prospective for the Functions of Sperm miRNAs

The idea that the ancestral environment can influence the phenotype of succeeding generations of offspring is similar in concept to the inheritance of acquired characteristics most famously championed by Jean-Baptiste Lamarck in the 19th century. This concept defines a potential mechanism for how organisms can adapt and then transmit characteristics important for adaption to their progeny by describing how the circumstances experienced by an organism could induce alterations in the next generations. This contrasts with Darwin’s theory of Natural Selection which postulates that organisms are predisposed with a range of traits and that those endowed with traits that provide increased survival and reproduction are naturally selected and propagated. However, Darwin also believed that the environment modulates the heritability of adaptive traits. This belief was captured in his concept of “Pangenesis” described in his 1868 work *The Variation of Animals and Plants under Domestication*. Darwin conceived of this hypothetical mechanism of heredity to explain the source of phenotypic variability prior to the discovery of genetic mutation and Mendelian inheritance. According to Darwin’s theory, various parts of the body that interact with the environment produce “gemmules” that are released from their tissue of origin and aggregate in the germline, contributing heritable information to gametes and subsequently to progeny upon fertilization.

In the early 20th century, these theoretical postulations of the environment modulating inherited information to confer adaptive advantages were laid to rest by August Weismann. The strict delineation between the immortal germline and perishable soma became known as the “Weismann barrier”. Weismann performed a series of experiments in mice where he shortened the tails of males and females, subsequently breeding them together and again shortening the tails for over twenty generations. Throughout this process, Weismann measured the tail length (prior to cutting) of the resulting progeny for every generation and observed no changes in natural tail length over subsequent generations. With this evidence, Weismann concluded that hereditary information contained in germ cells is uncompromised by parental exposures or experiences incurred by the soma. While this notion dominated preceding concepts of inheritance which have evolved over the 20th century, mechanisms that “break the Weismann barrier” have only recently been discovered [92]. With the discovery of the transfer of RNA from the epididymis to sperm together with the exponentially growing literature documenting that the parental environment can influence offspring phenotypes, there has been a recent revival of the Inheritance of Acquired Characteristics and “Lamarckian thought”. While it is clear that morphological insults to an animal, such as cutting a mouse’s tail off, do not transmit gross changes to their progeny, it is now widely accepted that more subtle phenotypes can be conferred by the ancestral environment.

Many publications document the regulation of sperm miRNAs upon changes to the paternal environment and demonstrate the non-genetic inheritance of environmentally modulated phenotypes in offspring (Table 1). While the causal demonstration of this phenomenon is still scarce, there are now several examples reviewed here where sperm miRNAs have been conclusively shown to transmit epigenetically inherited phenotypes to offspring. However, even in these examples, the mechanism for how a miRNA transmitted by sperm to the egg during fertilization can generate a phenotype in the developing progeny remains unclear. We propose that, at least initially, the sperm miRNA alters gene expression in the developing embryo shortly after fertilization. This alteration of gene expression could be induced by miRNA targeting of the 3′UTR of mRNAs that acts to either decrease transcript stability (thereby decreasing target mRNA levels) or regulating mRNA translation. The miRNA-mediated regulation of translation has yet to be identified for sperm miRNAs acting in the early embryo; however, miRNA regulation of gene expression in the embryo is unique compared to other physiological contexts. miRNAs in mice (including embryonic stem cells), zebrafish, frogs, and worm embryos have been shown to regulate gene expression preferentially via modulating translation as opposed to altering mRNA stability [93,94]. The regulation of embryonic mRNA stability by sperm RNAs has been demonstrated in several examples (described above), while others have computationally predicted the post-fertilization targets of sperm miRNAs. However, no direct, experimentally validated embryonic mRNA targets of sperm miRNAs have been identified to date. This gap in the literature can be largely attributed to the technical challenges of identifying direct miRNA targets experimentally, which currently rely on immunoprecipitation (IP) of AGO/miRNA/mRNA complexes and RNA-sequencing of bound substrates to determine the identity of targeted mRNAs. Recovery of complexes after IP is largely inefficient, requiring a large amount of input material, thereby preventing these types of studies from being performed in mammalian embryos. This technical limitation must be addressed with novel molecular techniques to identify the first regulatory functions initiated by sperm miRNAs in the early embryo.

Even after the targets of sperm miRNAs can be identified experimentally, several outstanding questions remain. To fully answer the question of how sperm miRNAs program offspring phenotype, the stoichiometric barrier of miRNA delivery to the embryo must be investigated. Mammalian eggs are typically hundreds to thousands of times larger in volume than their sperm counterparts. Thus, the contents of sperm, including miRNAs, are highly diluted upon fertilization. AGO2 and miRNAs bind their target mRNAs with a seven nt seed match with a measured equilibrium dissociation factor, K_d_, of about 10 pM [95,96]. Abundant miRNAs in sperm are present at between 100–10,000 copies per sperm, suggesting that it is possible that miRNAs bound to AGO are at levels sufficient to interact with their mRNA targets via their canonical regulation mechanism in the zygote even after dilution during fertilization [23]. Additionally, non-canonical miRNA functions in association with Argonautes 1, 3, and 4 have been demonstrated, including interaction with and regulation of chromatin [97]. Sperm miRNAs could potentially function via non-traditional mRNA targeting and regulatory mechanisms to program embryonic gene expression and development despite the drastic dilution that occurs during fertilization. Thus, it is important for the field to maintain an open perspective when thinking about the regulatory mechanisms employed by sperm miRNAs in the regulation of early embryogenesis.

Another consideration for determining the functions and targets of sperm miRNAs after fertilization is the pool of mRNAs that are regulated. These can be either the maternally deposited transcripts and/or the first transcripts transcribed during zygotic genome activation (ZGA). Sperm miRNAs could potentially regulate the stability or translation of maternal transcripts immediately after fertilization to the early two-cell stage of embryonic development. They could also potentially regulate newly transcribed zygotic mRNAs. The major wave of ZGA in mice occurs in the two-cell stage ~24 h after fertilization with smaller waves occurring earlier [98]. While the half-life or stability of sperm miRNAs after fertilization are completely unknown, it is reasonable to predict 24 h or more considering the half-life of miRNAs quantitively determined in other contexts [99]. Determining whether sperm miRNAs regulate maternal and/or newly synthesize zygotic transcripts would be informative from a functional perspective, as this would be a first step to understanding how their regulation can affect the first major transitions of early development. If maternal transcripts are regulated, this regulation may influence when, which, and to what extent specific genes are transcribed during ZGA triggering changes from the late two-cell stage onward via secondary effects of sperm miRNA regulation. Conversely, sperm miRNAs may affect mRNAs transcribed during ZGA and directly shape the stability or the translation of mRNAs as early embryonic progression occurs.

Finally, we must return to the prominent lingering question: How can a sperm miRNA generate an altered phenotype in a fully developed offspring? Answers to all the points of consideration highlighted above would shed light on the first steps of the process of epigenetic inheritance encoded by sperm miRNAs; however, it would still only reveal the tip of the iceberg. The early functions of sperm miRNAs in regulating embryonic gene expression must be perpetuated through preimplantation embryogenesis and later development in order to generate a fully developed phenotype. This must occur in absence of sperm miRNA as they are eventually turned over during early embryogenesis. How does this occur? It could potentially be programmed by the signal initiated by sperm miRNAs being perpetuated by a regulatory feedback-loop via miRNA and target mRNAs that are transcriptional regulators (or other regulators of gene expression). It could also occur by converting the regulatory signal into gene regulatory information that can be replicated during cell divisions by the establishment of chromatin domains which actively silence or license active gene expression. After the sperm miRNA signal is converted into a gene regulatory program that can be perpetuated throughout development, it must be considered whether this program can then be actively maintained and lost in specific cell types as the many cellular differentiation events required for development begin to occur. We hypothesize that, after fertilization, sperm miRNAs trigger a complex series of events in the embryo to regulate gene expression and the cascade of developmental processes that eventually produce an epigenetically transmitted physiological change generating an inherited phenotype. To say the very least, there is a lot for us to learn. We believe chipping away at the initial functions of sperm miRNAs in the regulation of gene expression and embryonic development are the first steps in undertaking the challenge of understanding the mechanisms underlying paternally transmitted epigenetic inheritance in mammals.

**Table 1:** A list of paternal environmental exposures and their effects on sperm miRNAs and progeny phenotypes. Experiments to determine the causality of sperm miRNAs regulated by the paternal environment in transmitting epigenetically inherited phenotypes to progeny are additionally noted. Males directly exposed to treatment are presented as the F_0_ generation in this review, and the offspring of these males mated with control/naive females the F_1_ generation. If applicable, F_2_ progeny were generated by mating F_1_ male offspring of exposed fathers to control females. Additional generations of progeny (F_3_, etc.) were made similarly.

	Experimental Design	Phenotype of Progeny	Dysregulated Sperm miRNAs	Phenotype of Offspring Produced from RNA Microinjection into Embryos	Citations
Diet/Metabolism (HFD)	Male Sprague–Dawley rats were fed HFD (42/45% energy from fat) for twelve weeks before mating.	When fed the control diet: F_1_ offspring displayed decreased body weight at three days of age compared to offspring of control rats *let-7c* was differentially expressed in metabolic tissues. F_2_ offspring had decreased birth weight and increased insulin levels during a glucose tolerance test compared to the control group. Both F_1_ and F_2_ progeny exhibited increased glucose tolerance when fed the control diet. When fed HFD: F_1_ and F_2_ generations displayed decreased *let-7c* levels in white adipose tissue. F_1_ and F_2_ females were resistant to HFD-induced weight gain compared to control groups with a 12–22% decrease in fat mass. Only F_2_ females showed impairments in glucose tolerance.	*let-7c* differentially expressed in both F_0_ fathers fed HFD and F_1_ sons fed control diet. *miR-293-5p* and *miR-880-3p* were downregulated and *let-7c-5p* was upregulated in both F_0_ and F_1_ generations compared to their respective controls.	Unconfirmed.	[69] de Castro Barbosa et al., 2016
Diet/Metabolism (HFD)	Male C57BL/6 mice were fed HFD (21% butterfat instead of 6%) starting at five weeks of age for ten weeks to induce obesity without diabetes. All progeny were raised on control diet and raised by their birth fathers.	F_1_ offspring displayed increased body weight from day five into adulthood. Male progeny had normal body composition, but plasma leptin was increased. Female progeny were obese throughout their lifespan, had increased adipose tissue depots, and increased circulating lipids at 17 weeks of age. F_1_ male offspring exhibited impaired glucose tolerance starting at eight weeks of age until 26 weeks. Insulin sensitivity was shown after 26 weeks of age. F_1_ female offspring also exhibited impaired glucose tolerance from eight weeks of age and impaired insulin sensitivity from 16 weeks of age. The F_2_ generation also displayed similar metabolic phenotypes.	F_0_ generation: *miR-133b*, *miR-196a-5p*, and *miR-205-5p* were upregulated. *miR-340-5p* was downregulated. F_1_ generation: *miR-126-3p*, *miR-135b*, *miR-143-3p*, *miR-133b-3p*, *miR-136-5p*, *miR-126- 5p*, *miR-141-5p*, *miR-145a-5p*, *miR-337-3p*, *miR-30a-5p*, and *miR-376a-3p* were upregulated. *miR-1961* and *miR-184-3p* were downregulated.	Unconfirmed.	[70] Fullston et al., 2013 [71] Fullston et al., 2016
Diet/Metabolism (HFD)	Male Sprague–Dawley rats were fed HFD (Lard-fed: 60% of total calories provided by lipids compared to 16% in control diet) from weaning at 21 days after birth to 12 weeks before mating. A carcinogen (DMBA) was given to 50-day old female offspring raised on control diet to induce mammary tumors.	Female offspring of lard-fed male rats exhibited greater birth weight and weight gain compared to control-fed offspring. Upon DMBA administration, female progeny of fathers fed lard-based diet had increased mammary tumor incidence, an increased number of proliferative cells, and fewer apoptotic cells in mammary gland lobules. Mammary tumors had fewer apoptotic cells.		Unconfirmed.	[72] Fontelles et al., 2016a
Diet/Met (HFD)	Extension of the experiment explained immediately above. Additional treatment group: Male rats were fed a lard diet consisting of 60% of total calories from corn oil.	Female offspring of corn oil-fed male rats exhibited less weight gain than offspring of lard-fed male rats and greater weight gain than their control counterparts. Female progeny of corn oil-fed rats had higher fasting glucose levels. Female progeny corn oil-fed fathers presented with decreased tumor latency and tumor growth and multiplicity compared to their lard diet counterparts. Mammary cells had fewer apoptotic cells.	Corn oil-fed fathers had 89 downregulated miRNAs compared to lard-fed rats. *miR-1897-5p*, *miR-219-1-3p*, and *miR-376a* were downregulated in both the corn oil-fed fathers’ sperm and the mammary glands of their female offspring.	Unconfirmed.	[72] Fontelles et al., 2016a
Diet/Met	Male C57BL/6 mice were fed an obesity-inducing diet (58% energy from fat compared to 17% in control) from three to ten weeks of age. A carcinogen (DMBA) was administered to induce mammary tumors.	Female offspring of male mice presented with higher birth weight that persisted through early adulthood, and their mammary tissues had more undifferentiated structures and were associated with increased incidence of DMBA-induced mammary tumors.	Three miRNAs, *miR-1896*, *miR-874*, and *miR-296-5p* were downregulated in both the sperm of fathers and their female offspring’s mammary tissue.	Unconfirmed.	[73] Fontelles et al., 2016b
Diet/Metabolism (WLD)	Male C57BL/6 mice were fed a Western-like diet consisting high sugar (34% sucrose) and high fat (21% butter).	Male and female offspring raised on standard diet had increased body weights. Male progeny had altered fasting blood glucose and higher glycemia in response to glucose and insulin injections.	*miR-182*, *miR-19a/b*, *miR-29a*, and *miR-340* were dysregulated in testis and sperm of fathers. *miR-19b* and *miR-29a* were the two most abundant of this subset.	Zygotes were injected with *miR-19b* (synthetic sense single-strand microRNA). The animals developed from these injections displayed increased body weights, no increase in fasting glucose levels, and variable glucose tolerance and insulin sensitivity compared to control. When the progeny of *miR-19b* injected embryos were mated to control females, some of these progeny (F_2_ generation) developed the full *miR-19b* phenotypes despite normal metabolic features of their progenitors.	[74] Grandjean et al., 2015
Diet/Metabolism (WLD)	C57BL/6J mice were fed a Western-like diet (WLD) consisting of high fat and high sugar (45% of energy from fat compared to control 5%) for three months. WLD feeding was maintained for five successive generations through the paternal lineage.	Successive paternal generations all fed WLD produced progeny with increasingly exacerbated overweight phenotype and accelerated obesity associated pathology development. Male and female F_1_ progeny fed control diet were heavier than control animals and had impaired glucose tolerance.	Unconfirmed.	Total RNA from the sperm of either the first or fifth consecutive animal fed HFD was microinjected into naive zygotes. Both groups: 12-week-old male progeny were heavier than control progeny and had altered glucose and insulin responses. Neither abnormal triglyceride levels nor histological abnormalities were observed in the liver. Overweight phenotypes and aberrant glucose responses were partially transmitted to the F_2_ and F_3_ generations. F_2_ generations did not have altered glucose and insulin responses. Total RNA from F_4_ HFD fed males: All progeny up to the F_3_ generation had altered glucose and insulin responses.	[75] Raad et al., 2021
Diet/Met	Male C57BL/6J mice were singly housed with or without access to a running wheel for twelve weeks. Progeny were either fed HFD (60% energy by fat) or control diet (10% energy by fat).	Progeny of males subjected to wheel-running for twelve weeks produced offspring that were more susceptible to the adverse effects of HFD (increased body weight, adiposity, impaired glucose tolerance, and elevated insulin levels).	Significantly differentially expressed sperm miRNAs included *miR-483-3p*, *miR-431*, *miR-221*, and *miR-21*.	Unconfirmed.	[76] Murashov et al., 2016
Diet/Met	Male C57BL/6 mice were fed control diet ad libitum were singly housed with access to a running wheel for four weeks. The control group was singly housed with no access to a running wheel. Offspring were phenotyped as juveniles or weaned and group housed.	Male progeny of runners displayed suppressed reinstatement of juvenile fear memory and reduced anxiolytic behavioral phenotypes.	*miR-190b* and *miR-19b-2* were increased and *miR-133a-1*, *miR-133a-2*, and *miR-455* were decreased in sperm of runners. In a validation study, *miR-455* was confirmed to be significantly decreased, while *miR-133a* was upregulated, and *miR-19b* was downregulated. *miR-190b* displayed a non-significant decrease.	Unconfirmed.	[77] Short et al., 2017
Stress (MSUS)	C57BL/6J mice were subjected to three hours of proximal separation from their mothers from postnatal days one through fourteen. This model of unpredictable maternal separation combined with unpredictable maternal stress (MSUS) was used to expose mice to traumatic stress in early life. F_0_ MSUS mice had reduced avoidance and fear as shown by an elevated plus maze, altered response to aversive conditions as shown by a light–dark box, and altered behavioral despair as shown by a Porsolt forced swim test.	F_1_ offspring displayed the same behavioral phenotypes as their fathers, but also presented with more exacerbated metabolic phenotypes. These mice had lower insulin and glucose levels, normal glucose at baseline, but lower glucose rise on GTT and normal glucose decrease on ITT indicating insulin hypersensitivity. F_1_ progeny exhibited hypermetabolism. *miR-375* target, *Catenin β1*, was decreased in the hippocampus.	MSUS sperm displayed upregulation of *miR-375-3p*, *miR375-5p*, *miR-200b-3p*, *miR-672-5p*, and *miR-466-5p*. These miRNAs were also altered in serum, hippocampus, and hypothalamus of F_1_ offspring.	Total RNA from sperm was purified and microinjected into wild-type fertilized zygotes recapitulating the behavioral and metabolic phenotypes of progeny of MSUS treated male mice. The offspring sired by zygotically-injected mice mated to control females displayed similar depressive-like behaviors.	[81] Gapp et al., 2014
Stress (CVS)	Male C57BL/6:129 F_1_ hybrid mice (which provide reproducible balance of stress responsivity, behavioral performance, and maternal care) were exposed to six weeks of chronic variable stress through puberty or adulthood.	Both male and female progeny of stressed mice displayed lower corticosterone levels in response to restraint stress compared to progeny of unstressed control mice.	*miR-193-5p*, *miR-204*, *miR-29c*, *miR-30a/c*, *miR-32*, *miR-375*, *miR-532-3p*, and *miR-698* were significantly increased in both pubertal stress and adult stressed sperm.	Duplex miRNA mimics of *miR-193-5p*, *miR-204*, *miR-29c*, *miR-30a*, *miR-30c*, *miR-32*, *miR-375*, *miR-532-3p*, and *miR-698* were injected into zygotes fertilized by naive sperm recapitulated the offspring stress dysregulation phenotype and induced long-term reprogramming of the hypothalamic transcriptome with HPA axis dysfunction. In miRNA injected embryos, target mRNAs *Sirt1*, *Ube3a*, *IL6st*, *Ncl*, *Aars*, *Agfg1*, and *Ralbp1* were significantly reduced. *Ube3a* and *Sirt1* are involved in chromatin remodeling and neurodevelopmental disorders.	[82] Rodgers et al., 2013 [83] Rodgers et al., 2015
Stress (CVS)	Male C57BL/6:129 F_1_ hybrids were exposed to psychological stress daily for four weeks then mated to control F_1_ hybrid females at either nine or twenty weeks.	Offspring exposed to twelve weeks of stress exhibited HPA axis dysregulation as previously demonstrated. Broad expression patterns of sperm miRNAs from offspring were significantly altered after being exposed to twelve weeks of stress but not after one week exposure.		Unconfirmed. Naive sperm incubated with EVs isolated from in vitro culture of epididymal epithelial cells treated with corticosterone were used for ICSI. miRNA profiles in these cells changed in a pattern similar to the changes seen in stressed sperm compared to control sperm fertilized oocytes.	[48] Chan et al., 2020
Stress (CMS)	Male C57BL/6J mice were subjected to chronic mild stress daily for five weeks resulting in depression-like behaviors and decreased weight gain.	Progeny had normal phenotypes under baseline conditions, but when exposed to slight chronic variable stress for two weeks, they presented with depressive-like phenotypes and increased plasma corticosterone levels. Progeny not exposed to stress had similar synaptic transmission and neuronal activity profiles in the neural circuits as their stressed fathers.	Sixteen miRNAs were upregulated, and one miRNA was downregulated in sperm of stressed fathers. *miR-146a-5p*, *miR-27b-3p*, *miR-30a-5p*, *miR-152-3p*, *miR-1839-5p*, *miR-143-3p*, *miR-9-5p*, *let-7g-5p*, *miR-200a-3p*, *miR-200c-3p*, *miR-30c-5p*, *miR-26b-5p*, *miR-103-3p*, *miR-29a-3p*, *miR-101a-3p*, and *miR-199a-3p* were upregulated, and *miR-184-3p* was downregulated. *miR-191-5p* and *miR-30a-3p* were upregulated in sperm exposed to stress but were not confirmed by RT-qPCR. *App*, *Tspan7*, *Wnk3*, *Ly6a*, *Grin3a*, and *βCamkII* transcript levels were decreased and found to be targeted by dysregulated miRNAs in embryonic stem cells transfected with synthetic miRNA mimics. Direct binding was confirmed by a luciferase reporter assay. In embryos injected with small RNAs from stressed sperm, *App*, *Tspan7*, *Wnk3*, *Ly6a*, and *Grin3a* levels were decreased from the four-cell stage to the morula stage, while *βCamkII* expression was increased.	Aberrant phenotypes were recapitulated after injection of synthetic miRNA duplexes mimicking the sixteen most highly expressed (upregulated) miRNAs in sperm into naive zygotes.	[84] Wang et al., 2021
Stress (CORT)	Ten-week-old male C57BL/6 mice were given corticosterone-supplemented water (25 ug/mL) for four weeks ad libitum.	F_1_ female offspring displayed changes in changes in early-life fear conditioning with no changes to behavior in adulthood. F_1_ male offspring exhibited altered anxiety-related behaviors and a significant increase in *Igf*_2_ mRNA expression in the hippocampus at eight weeks of age. F_2_ progeny displayed selective behavioral effects. Both male and females spend more time in the open arms of the elevated-plus maze. Only male progeny had increased hippocampus *Igf*_2_ mRNA expression.	Forty-six miRNAs were changed by two-fold or greater in CORT sperm compared to control. The top 20 ranked miRNAs were analyzed to find predicted downstream gene targets. The top miRNAs regulated included: *miR-144*, *miR-190b*, *miR-192*, *miR-26b*, *miR-350*, *miR-449a*, *miR-467e*, and *miR-98*. *miR-98*, *miR-144*, and *miR-190b* were validated as highly expressed in the sperm of a separate cohort of CORT-treated mice. While no significant change in the expression of *miR-192* or *miR-449a* were found. CORT treated samples had significant variability compared to controls.	Unconfirmed.	[85] Short et al., 2016
Stress	Sperm from Caucasian men (average age 32.4 years) exposed to stress in early life as identified by the adverse childhood experience (ACE) questionnaire were analyzed for changes in miRNA content.		miRNAs in the *miR-34/449* family were the most significantly dysregulated.	Unconfirmed.	[87] Dickson et al., 2018
Stress (CSI)	Male CD-1 (outbred) strain mice were exposed to chronic social instability (CSI) stress onset 28 days postnatal age for seven weeks.	F_1_ female offspring exhibited anxiety and sociability defects. F_1_ male offspring had no anxiety or sociability defects. F_2_ female offspring had anxiety and sociability behavioral defects.	*miR-449a* and *miR-34c* were decreased in CSI sperm compared to control. *miR-449* and *miR-34* have the same seed sequence. Knockout mice of these miRNAs display defects in brain development and spermatogenesis caused in part by defective microtubule and cilia function. Targets of these miRNAs include *p53*, *CDK6*, *c-MYC*, *HDAC1*, and *BCL-2*, which are all strong regulators of gene expression and development.	Unconfirmed.	[87] Dickson et al., 2018
Toxins (POPs)	Male Sprague–Dawley rats were exposed to persistent organic pollutants (POPs) or corn oil control in utero four weeks before gestation and until parturition.	The progeny of POPs exposed males presented with multiple developmental and disease conditions including neurodevelopmental deficits, altered reproductive functions, and immunotoxicity.	The F_0_ generation had ten upregulated sperm miRNAs (*miR-466c-5p*, *miR-471-5p*, *miR-194-5p*, *miR-17-5p*, *miR-374-5p*, *miR-30b-5p*, *miR-296-5p*, *miR-19b-3p*, *miR-6334*, and *miR-32-5p*). The F_1_ generation had 37 dysregulated miRNAs in their sperm, F_2_ had ten, and F_3_ had one. *miR-6334*, *miR-19b-3b*, and *miR-30b-5p* were intergenerationally dysregulated.	Unconfirmed.	[88] Herst et al., 2019
Toxins	Extension of the immediately above experiment. Additional treatment group: Male rats exposed to POPS were also treated with folic acid (FA) supplementation in utero.	FA supplementation males produced progeny with fewer miRNAs regulated in their sperm compared to POPs exposed male progeny.	The F_0_ generation only had one upregulated miRNA, *miR-6334*. The F_1_ generation had twelve upregulated miRNAs. Only *miR-6334* was affected across multiple generations.	Unconfirmed.	[88] Herst et al., 2019
Toxins (Vin)	Female Hsd:Sprague–Dawley (outbred) rats were administered daily IP injections of vinclozolin (100 mg/kg BW/day) on days eight through fourteen of gestation.		Two hundred and twenty-two sncRNAs were significantly differentially expressed in vinclozolin lineage F_3_ generation sperm compared to control sperm. Thirteen miRNAs were upregulated and eight were downregulated.	Unconfirmed.	[89] Schuster et al., 2016
Toxins (Smoke)	Seven non-smokers and six smokers’ semen samples were analyzed for miRNA changes using microarray.		*miR-365*, *miR-944*, *miR-1267*, *miR-340*, *miR-4513*, *let-7a-2-3p*, *miR-576-3p/5p*, *miR-1246*, *miR-30c*, *miR-933*, *miR-7*, *miR-1285*, *miR-1270*, *miR-509-5p*, *miR-146b-3p*, *miR-3145-3p*, *miR-4748*, *miR-519d*, and *miR-550a/b* were upregulated. *miR-574-5p*, *miR-3145-5p*, *miR-146b-5p*, *miR-634*, *miR-129-3p*, *miR-652*, and *miR-4723-5p* were downregulated.	Unconfirmed.	[90] Marczylo et al., 2012
Toxins (Ethanol)	Eight-week-old male C57BL/6J mice were exposed to ethanol inhalation chambers in their home cage for five weeks, eight hours a day, and five days a week. Animals were group-housed. Sperm were collected from cauda epididymis 24 h after the final ethanol or control exposure.		*miR-10a*, *miR-99b*, *miR-3535*, *miR-196a*, *miR-205*, *miR-125a*, and *miR-16a* were upregulated, and only one miRNA was significantly downregulated. The same sperm sncRNAs altered in sperm are also altered in epididymosomes.	Unconfirmed.	[91] Rompala et al., 2018
Toxins (Ethanol)	Ten-week-old male C57BL/6J mice treated in either ethanol inhalation chambers or control chambers were sacrificed for epididymosome collection. Sperm from control mice were collected and incubated with epididymosomes from either ethanol-treated or control mice then used for IVF with eggs collected from superovulated six-week-old female C57BL/6J mice. Embryos were cultured until the two-cell stage at which point they were transferred to pseudopregnant CD-1 foster mothers for full-term development.	Adult male progeny of sperm incubated with ethanol epididymosomes presented with altered body weight and modestly altered binge ethanol drinking compared to control sperm while adult female progeny displayed basal anxiety-like behavior and sensitivity to an anxiolytic dose of ethanol.	Unconfirmed.	Unconfirmed.	[47] Rompala et al., 2020
